# Hospital Variation in Preference for a Specific Bariatric Procedure and the Association with Weight Loss Performance: a Nationwide Analysis

**DOI:** 10.1007/s11695-022-06212-8

**Published:** 2022-09-14

**Authors:** Erman O. Akpinar, Ronald S. L. Liem, Simon W. Nienhuijs, Jan Willem M. Greve, Perla J. Marang-van de Mheen, on behalf of the Dutch Audit for Treatment of Obesity Research Group, L. M. de Brauw, S. M. M. de Castro, S. L. Damen, A. Demirkiran, M. Dunkelgrün, I. F. Faneyte, G. van ’t Hof, I. M. C. Janssen, E. H. Jutte, R. A. Klaassen, E. A. G. L. Lagae, B. S. Langenhoff, A. A. P. M. Luijten, R. Schouten, R. M. Smeenk, D. J. Swank, M. J. Wiezer, W. Vening

**Affiliations:** 1 Department of Surgery, NUTRIM School for Nutrition and Translational Research in Metabolism, Maastricht University Medical Center, 6229 HX Maastricht, the Netherlands; 2 Scientific Bureau, Dutch Institute for Clinical Auditing, Leiden, the Netherlands; 3 Department of Surgery, Groene Hart Hospital, Gouda, the Netherlands; 4 Dutch Obesity Clinic, The Hague & Gouda, the Netherlands; 5 Department of Surgery, Catharina Hospital, Eindhoven, the Netherlands; 6 Department of Surgery, Zuyderland Medical Center, Heerlen, the Netherlands; 7 Dutch Obesity Clinic South, Heerlen, the Netherlands; 8 Department of Biomedical Data Sciences, Medical Decision Making, Leiden University Medical Center, Leiden, the Netherlands

**Keywords:** Hospital preference, Hospital variation, Hospital volume, Centralized bariatric care, Weight loss, Bariatric surgery, Textbook outcome

## Abstract

**Purpose:**

Hospitals performing a certain bariatric procedure in high volumes may have better outcomes. However, they could also have worse outcomes for some patients who are better off receiving another procedure. This study evaluates the effect of hospital preference for a specific type of bariatric procedure on their overall weight loss results.

**Methods:**

All hospitals performing bariatric surgery were included from the nationwide Dutch Audit for Treatment of Obesity. For each hospital, the expected (E) numbers of sleeve gastrectomy (SG), Roux-en-Y gastric bypass (RYGB), and one-anastomosis gastric bypass (OAGB) were calculated given their patient-mix. These were compared with the observed (O) numbers as the O/E ratio in a funnel plot. The 95% control intervals were used to identify outlier hospitals performing a certain procedure significantly more often than expected given their patient-mix (defined as hospital preference for that procedure). Similarly, funnel plots were created for the outcome of patients achieving ≥ 25% total weight loss (TWL) after 2 years, which was linked to each hospital’s preference.

**Results:**

A total of 34,558 patients were included, with 23,154 patients completing a 2-year follow-up, of whom 79.6% achieved ≥ 25%TWL. Nine hospitals had a preference for RYGB (range O/E ratio [1.09–1.53]), with 1 having significantly more patients achieving ≥ 25%TWL (O/E ratio [1.06]). Of 6 hospitals with a preference for SG (range O/E ratio [1.10–2.71]), one hospital had significantly fewer patients achieving ≥ 25%TWL (O/E ratio [0.90]), and from two hospitals with a preference for OAGB (range O/E ratio [4.0–6.0]), one had significantly more patients achieving ≥ 25%TWL (O/E ratio [1.07]). One hospital had no preference for any procedure but did have significantly more patients achieving ≥ 25%TWL (O/E ratio [1.10]).

**Conclusion:**

Hospital preference is not consistently associated with better overall weight loss results. This suggests that even though experience with a procedure may be slightly less in hospitals not having a preference, it is still sufficient to achieve similar weight loss outcomes when surgery is provided in centralized high-volume bariatric institutions.

**Graphical Abstract:**

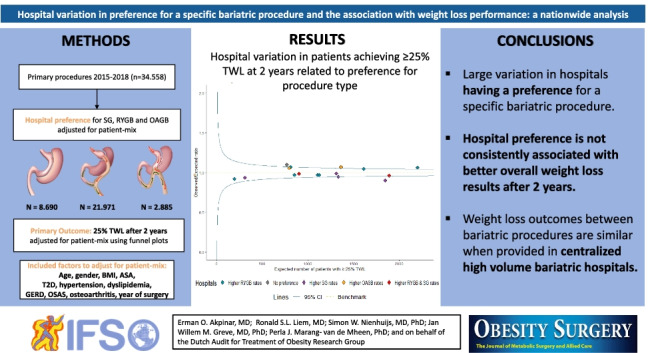

**Supplementary Information:**

The online version contains supplementary material available at 10.1007/s11695-022-06212-8.

## Introduction

To effectively treat patients with morbid obesity, a variety of bariatric surgical procedures are available. Literature has extensively demonstrated the effectiveness of bariatric procedures in terms of weight loss and comorbidity reduction, but each procedure will have its own advantages considering some outcomes, while having disadvantages in terms of other outcomes [1–5]. This makes it crucial to tailor the best procedure to the characteristics of individual patients, e.g., performing a Roux-en-Y gastric bypass (RYGB) for patients with gastro-esophageal reflux disease (GERD) [6].

However, surgeon preference may also play a significant role in decision making around type of bariatric procedure [7]. Factors relevant for shared decision making are weight loss outcomes, patients’ preference, and reduction of relevant comorbidities such as GERD or type 2 diabetes (T2D) [8]. Furthermore, bariatric surgery has a history of trends with frequent changes in techniques and procedures [9]. Nowadays, the most frequently performed procedures are the one-anastomosis gastric bypass (OAGB), RYGB, and the sleeve gastrectomy (SG), with SG being the world’s predominant procedure due to lower long-term morbidity and similar weight loss results as RYGB [2, 9–12].

These trends and changes in bariatric surgery have led to different physician preferences, with many surgeons predominantly performing one procedure [12]. Previous studies have shown that high operative volume of a single procedure is associated with lower morbidity[13, 14], consistent with the notion “Practice makes perfect.” Having extensive experience with one specific technique in a high volume center could therefore result in better overall hospital outcomes. On the other hand, a one-size-fits-all policy may also result in worse outcomes for some patients who, based on their patient characteristics, would be better off with a different type of bariatric procedure.

Therefore, the present study will evaluate the extent to which hospitals perform some specific bariatric procedures more than expected given their patient-mix, and whether such hospital preference in high volume centers is associated with overall hospital performance on patients achieving 25% total weight loss (TWL) after 2 years.

## Materials and Methods

### Setting and Study Design

In the Netherlands, bariatric surgical care is centralized in hospitals since 2010, using rather uniform peri- and postoperative care protocols [15]. All hospitals perform bariatric surgery with a multidisciplinary team, including at least 2 dedicated bariatric surgeons and performing at least 200 procedures annually. This minimum number of annual procedures is based on the Dutch guidelines to ensure high surgeon experience on an institutional level. All included hospitals in the current study have at least 2 dedicated bariatric surgeons performing a minimal of 200 procedures annually for at least 5 years [15].

Data were derived from the nationwide quality registry DATO (Dutch Audit for Treatment of Obesity) [16]. The present study was approved by all scientific committee members of the DATO and has been performed following the ethical standards stated in Dutch law. The DATO is an opt-out quality registry with anonymized data which cannot be traced back to the individual patient, so that according to applicable Dutch regulations, no informed consent was needed for this study.

### Patient Selection

All patients who underwent a primary SG, RYGB, or OAGB between 2015 and 2018 were included in the analysis. To evaluate the current Dutch situation, patients were excluded if they underwent bariatric surgery in hospitals that stopped performing bariatric surgery. Therefore, we included all 16 hospitals that performed bariatric surgery from 2015 to the present; 2 hospitals that stopped treating bariatric patients in this period were excluded. Patients with missing data on date of birth, weight, length, obesity related comorbidities during preoperative screening, or procedure type were excluded.

### Definitions and Outcome Parameters

The choice for a specific bariatric procedure should be tailored based on the individual patient’s characteristics. Therefore, hospital preference for a specific bariatric procedure was defined as performing significantly more of this specific procedure than would be expected based on the patient-mix treated in that hospital. The calculation of expected numbers is explained in more detail in the statistical analysis section. The following patient characteristics were taken into account: age, sex, body mass index (BMI), American Society of Anesthesiologists (ASA) classification, year of operation, GERD, T2D, hypertension, obstructive sleep apnea syndrome (OSAS), dyslipidemia, and osteoarthritis, which were defined as described previously [17].

The primary outcome is patients achieving ≥ 25% TWL (total weight loss) i.e., for all patients in a hospital after 2-year follow-up. Although 20% TWL is a common threshold for successful weight loss, 25% TWL was chosen from the perspective of hospitals continuously improving their care, which is better supported by a threshold that is more discriminative as shown by a previous study [18]. The nationally predefined interval for a follow-up at 2 years is an outpatient clinic visit between 21 and 27 months postoperatively. Total weight loss at 2-year follow-up is defined as: $\frac{(preoperative\:weight-followup\:weight)}{preoperative\:weight}$  * 100% = *% TWL.* Secondary outcome was the composite measure Textbook Outcome, which is defined as: no mortality, no severe postoperative complications, no readmissions, no mild complications, and no prolonged length of stay (LOS) (> 2 days) within 30 days after primary bariatric surgery [19]. This was chosen because it provides additional insight in the direct postoperative quality of care delivered by the hospital, from the rationale that if practice makes perfect, hospitals with a preference for a specific type of procedure might have better Textbook Outcome.

### Statistical Analysis

Baseline characteristics were compared between patients undergoing different types of bariatric procedures, using descriptive statistics. Pearson Chi-square test was used to compare categorical variables and the analysis of variance (ANOVA) for continuous variables.

Subsequently, nationwide hospital variation was evaluated in their preference to perform a specific bariatric procedure more often than would be expected given their patient-mix, using a funnel plot. First, multivariable logistic regression was performed using data from all patients in all hospitals, to estimate the extent to which certain characteristics made it more or less likely for the patient to undergo a specific bariatric procedure. All of the aforementioned patient characteristics were included as independent variables based on literature[20] and clinical relevance, and undergoing a specific bariatric procedure (yes/no) as the dependent variable. This was done separately for each of the three bariatric procedures. The coefficients from these models were used to estimate for each patient the expected probability to undergo each of the three bariatric procedures based on patient characteristics. These probabilities were summed across patients within each hospital to arrive at the aggregated expected number (E) of specific bariatric procedures performed in that hospital. The observed number (O) of specific bariatric procedures was then divided by the expected number for that hospital to calculate the O/E ratio [21]. Subsequently, we graphically plotted all hospitals with their O/E ratios in a patient-mix adjusted funnel plot along with 95% Control Intervals (CI). Hospitals above the upper 95%CI performed significantly more of a specific bariatric procedure than expected based on their patient-mix and were defined as having a preference for that bariatric procedure. Hospitals under the lower 95%CI were significantly less likely to perform that particular procedure, which likely meant they had preference for another procedure and were therefore not further described. Hospitals in between the 95%CI were performing as expected given their patient-mix and were defined as having no specific preference. The funnel plot inherently takes into account differences in absolute numbers of procedures. This difference is shown by the funnel shape of the control interval, which is broader for hospitals with lower numbers and narrower for hospitals with higher absolute numbers, meaning that a smaller preference can be identified as significantly different for hospitals with higher absolute numbers.

Similarly, patient-mix adjusted funnel plots were created for the primary outcome of patients achieving ≥ 25% TWL after 2 years, including the same patient characteristics. All hospitals were color coded depending on their preference for a specific bariatric procedure. If hospitals had a preference for more than one procedure, they were given a separate color to indicate preference for the combination rather than counted by both types of procedures. All statistical analyses were performed in R version 3.4.2.

### Sensitivity Analysis

Short-term weight loss results at 1-year follow-up have shown to be similar across bariatric procedures. Although 2-year follow-up was assessed, it may not have been long enough to show the impact on weight loss. Therefore, a sensitivity analysis was conducted including all patients undergoing bariatric surgery in 2015 with a complete 5-year follow-up to examine the association of hospital preference for a specific bariatric procedure and long-term weight loss. Hospital preference from the main analyses was used, based on all patients. Patient-mix adjusted funnel plots were created to show hospital performance on patients achieving ≥ 25% TWL after 5 years, including all aforementioned patient characteristics.

## Results

### Study Sample

Between 2015 and 2018, 34,866 patients underwent a primary bariatric procedure of whom 34,558 (99.1%) had complete data and were included for analysis. Hospitals had a median annual volume of 499 procedures (IQR 377–762). The follow-up at 2 year was 67% (*n* = 23.154), with limited hospital variation (median 70.2% [IQR 63.5–72.5%]). Table 1 shows significant differences in all baseline characteristics between patients undergoing RYGB, SG, OAGB, or another procedure, which emphasizes the need for patient-mix adjustment when comparing hospitals on the extent to which they perform certain procedures and their performance on patients achieving ≥ 25% TWL after 2 years.

**Table 1:** Patient characteristics of patients who underwent a primary bariatric procedure between 2015 and 2018

Characteristics	Type of procedure				*p* value
	RYGB	SG	OAGB	Others*	
*n*	21,971	8690	2885	1012	
Sex, no. (%)					
Male	4190 (19.1)	2230 (25.7)	736 (25.5)	249 (24.6)	< 0.01
Female	17,781 (80.9)	6460 (74.3)	2149 (74.5)	763 (75.4)	
Age, mean(SD)	44.66 (11.01)	41.77 (12.47)	45.66 (11.47)	44.39 (11.30)	< 0.01
BMI mean (SD)	43.23 (4.89)	45.44 (6.48)	46.07 (6.01)	43.05 (5.82)	< 0.01
ASA classification, no. (%)					
I–II	12,620 (57.4)	3822 (44.0)	985 (34.1)	523 (51.7)	< 0.01
≥ III	9351 (42.6)	4868 (56.0)	1900 (65.9)	489 (48.3)	
T2D, no. (%)					
Not present	17,180 (78.2)	7265 (83.6)	2135 (74.0)	798 (78.9)	< 0.01
Present	4791 (21.8)	1425 (16.4)	750 (26.0)	214 (21.1)	
Hypertension, no. (%)					
Not present	14,166 (64.5)	5910 (68.0)	1726 (59.8)	654 (64.6)	< 0.01
Present	7805 (35.5)	2780 (32.0)	1159 (40.2)	358 (35.4)	
Dyslipidemia, no. (%)					
Not present	17,168 (78.1)	7225 (83.1)	2349 (81.4)	812 (80.2)	< 0.01
Present	4803 (21.9)	1465 (16.9)	536 (18.6)	200 (19.8)	
GERD, no. (%)					
Not present	18,528 (84.3)	7640 (87.9)	2510 (87.0)	871 (86.1)	< 0.01
Present	3443 (15.7)	1050 (12.1)	375 (13.0)	141 (13.9)	
OSAS, no. (%)					
Not present	17,812 (81.1)	7081 (81.5)	2289 (79.3)	874 (86.4)	< 0.01
Present	4159 (18.9)	1609 (18.5)	596 (20.7)	138 (13.6)	
Osteoarthritis, no. (%)					
Not present	11,312 (51.5)	4911 (56.5)	2066 (71.6)	390 (38.5)	< 0.01
Present	10,659 (48.5)	3779 (43.5)	819 (28.4)	622 (61.5)	

*RYGB* Roux-en-Y gastric bypass, *SG* sleeve gastrectomy, *OAGB* one anastomosis gastric bypass, *BMI* body mass index, *ASA* American Society of Anesthesiologists, *T2D* type 2 diabetes mellitus, *GERD* gastro esophageal reflux disease, *OSAS* obstructive sleep apnea syndrome, *SD* standard deviation

^*^The group of “Other” procedures consists of gastric banding *n* = 91 (9%), BPD *n* = 4 (0.4%), SADI *n* = 32 (3.2%), banded gastric bypass *n* = 851 (84%), and other procedures *n* = 34 (3.4%)

### Hospital Preference

**Fig. 1:**
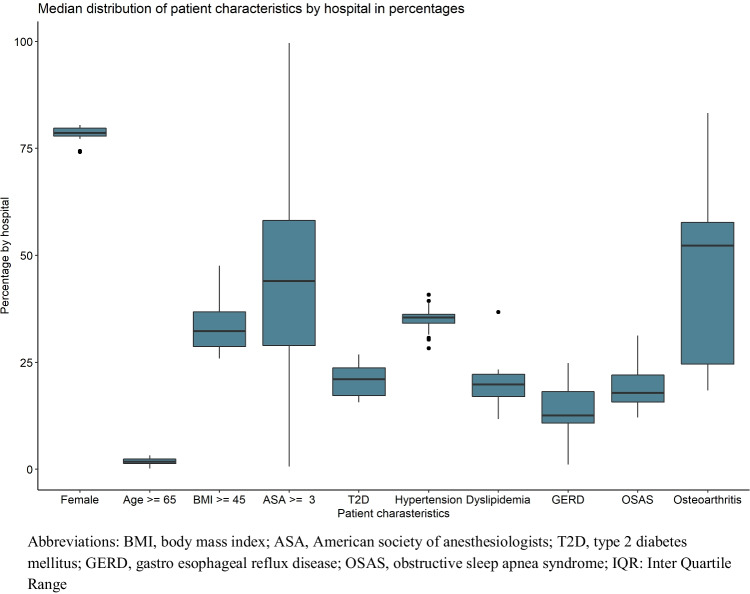
Boxplot showing the distribution of the median percentage (IQR) of patient characteristics by hospital in the Netherlands

The between-hospital variation in their patient-mix is shown in Fig. 1. Hospitals varied significantly in distribution for all patient-mix variables, but in particular for the percentage of patients with ASA ≥ 3 (median of 47.8% [IQR = 29.2–56.2%]), GERD (13% [IQR = 11.2–18.7%]), and osteoarthritis (51.6% [IQR = 24.5–57.1%]) at baseline.

**Fig. 2:**
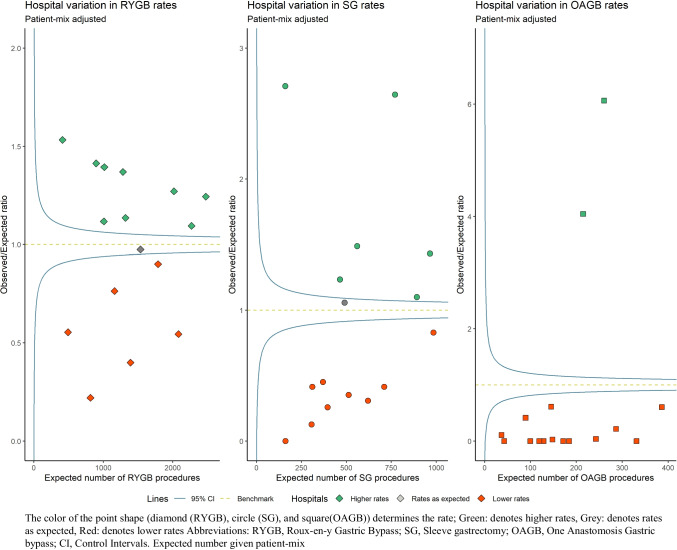
Patient-mix adjusted funnel plot showing hospital variation in preference for RYGB, SG, and OAGB procedures

Figure 2 shows the extent to which hospitals performed more RYGB, SG, or OAGB than expected based on their patient-mix, suggesting a preference for that specific procedure (depicted in green). Table 2 shows the extent to which patient characteristics influenced the odds to undergo a specific bariatric procedure. Female elderly patients with T2D, GERD, dyslipidemia, or osteoarthritis at baseline were more likely to undergo RYGB. Patients with higher BMI, higher ASA classification, and hypertension or osteoarthritis at baseline were more likely to undergo SG, and elderly patients with higher BMI, higher ASA classification, with T2D or hypertension at baseline were more likely to undergo OAGB. Nine hospitals performed significantly more RYGB (range in O/E ratio 1.09–1.53), six hospitals performed significantly more SG (range in O/E ratio 1.10–2.71), and 2 hospitals performed significantly more OAGB than expected given their patient-mix (range in O/E ratio 4.0–6.0). The hospitals indicated by a red color were significantly less likely to perform that type of procedure given their patient-mix, which could mean they had a preference for another type of procedure. Hospitals were indicated by a grey color if they performed as many procedures as would be expected given their patient-mix.

**Table 2:** Multivariable logistic regression analyses including all patients from all hospitals for undergoing a specific bariatric procedure based on patient-mix

Multivariable analyses	RYGB	SG	OAGB
	aOR [95% CI]	aOR [95% CI]	aOR [95% CI]
Sex			
Male	ref	ref	ref
Female	1.54 [1.46–1.63]	0.66 [0.62–0.7]	0.9 [0.82–0.98]
Age	1.01 [1.01–1.02]	0.98 [0.97–0.98]	1.02 [1.01–1.02]
BMI	0.94 [0.94–0.94]	1.05 [1.05–1.06]	1.06 [1.05–1.06]
ASA			
I/ II	ref	ref	ref
≥ III	0.56 [0.54–0.59]	1.55 [1.47–1.63]	1.85 [1.71–2.01]
T2D			
Not present	ref	ref	ref
Present	1.14 [1.07–1.21]	0.75 [0.7 –0.81]	1.23 [1.11–1.36]
Hypertension			
Not present	ref	ref	ref
Present	0.9 [0.85–0.95]	1.09 [1.03–1.16]	1.12 [1.02–1.22]
GERD			
Not present	ref	ref	ref
Present	1.16 [1.08–1.24]	0.85 [0.79–0.92]	1.00 [0.89–1.13]
Dyslipidemia			
Not present	ref	ref	ref
Present	1.15 [1.07–1.22]	0.96 [0.9–1.04]	0.76 [0.68–0.85]
OSAS			
Not present	ref	ref	ref
Present	1.04 [0.97–1.1]	1.04 [0.97–1.11]	0.99 [0.89–1.1]
Osteoarthritis			
Not present	ref	ref	ref
Present	1.1 [1.05–1.15]	1.08 [1.03–1.14]	0.44 [0.41–0.49]

*RYGB* Roux-en-Y gastric bypass, *SG* sleeve gastrectomy, *OAGB* one-anastomosis gastric bypass, *BMI* body mass index, *ASA* American Society of Anesthesiologists, *T2D* type 2 diabetes, *GERD* gastro esophageal reflux disease, *OSAS* obstructive sleep apnea syndrome, *aOR* adjusted odds ratio, *CI* confidence interval

### Association of Hospital Preference with Outcomes

**Fig. 3:**
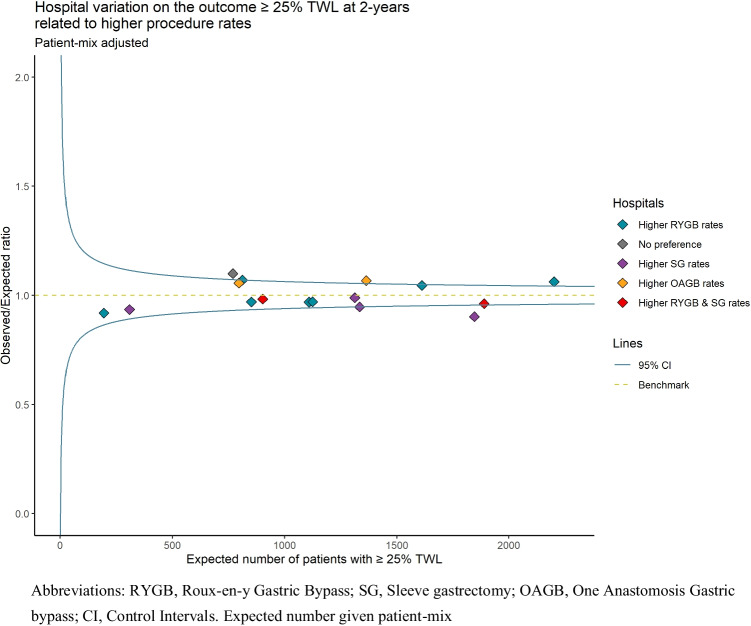
Patient-mix adjusted funnel plot showing hospital variation in 25% TWL after 2 years related to preference for type of procedure

Figure 3 shows how the preference for a particular type of bariatric surgery is associated with the overall hospital performance of patients achieving ≥ 25% TWL after 2 years. Most hospitals have a preference for one type of bariatric surgery, except for 1 hospital (in grey) without any preference, and 2 hospitals with a preference for both RYGB and SG. From the 9 hospitals with a preference for RYGB, one hospital had significantly more patients achieving ≥ 25% TWL after 2 years, i.e., better overall outcomes (O/E ratio 1.06), and one of the two hospitals with a preference for OAGB (O/E ratio 1.07). On the other hand, from the 6 hospitals with a preference for SG, one hospital had significantly worse overall outcomes as fewer patients achieved ≥ 25% TWL after 2 years (O/E ratio 0.90). The hospital (grey) with no preference for either RYGB, SG, or OAGB shows significantly better overall outcomes on ≥ 25% TWL after 2 years (O/E ratio 1.10). There were no significant differences between hospitals in the outcome ≥ 50% Excess Weight Loss (EWL) after 2 years (range O/E ratio 0.95–1.05).

**Fig. 4:**
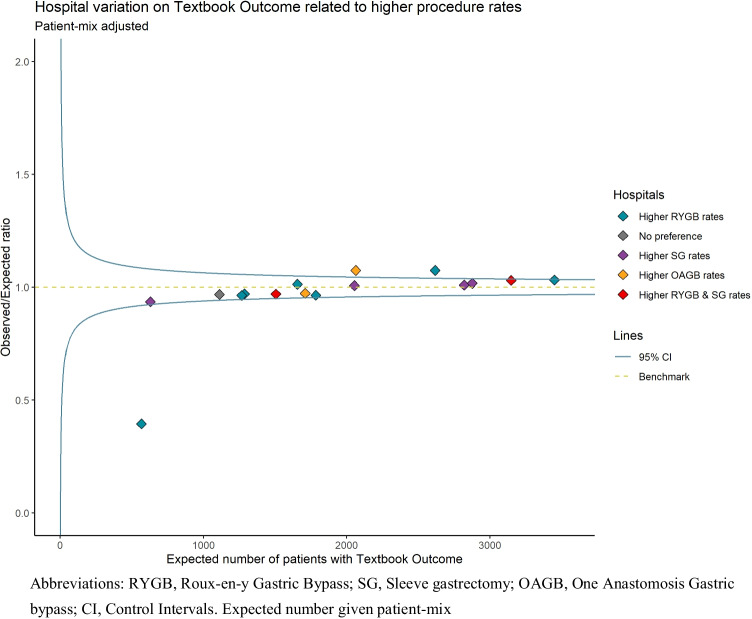
Patient-mix adjusted funnel plot showing hospital variation on textbook outcome related to preference for type of procedure

Figure 4 presents the between-hospital variation to achieve Textbook Outcome associated with hospital preference for a bariatric procedure. From the 9 hospitals with preference for RYGB, one hospital had significantly fewer patients achieving Textbook Outcome, i.e., worse performance (O/E ratio 0.40), and one hospital had significantly better performance (O/E ratio 1.07). One hospital with preference for OAGB had significantly better overall performance in patients achieving Textbook Outcome (O/E ratio 1.07). The remaining 13 hospitals all had a performance as expected in patients achieving Textbook Outcome. Looking specifically at postoperative severe complications, there was no association between hospital preference for a specific procedure and the percentage of Clavien Dindo ≥ III complications within 30 days (data not shown).

### Sensitivity Analysis

The follow-up at 5-years was 35.4% (*n* = 2565) with limited hospital variation (median 33.2% [IQR = 28.4–41.3%]). Even though the funnel plot has lower power to detect differences in hospital performance, as shown by wider control intervals, Supplemental Fig. 1 shows a very similar pattern for 5-year weight loss results as shown in Fig. 3 for 2-year weight loss results. Hospital preference for a specific bariatric procedure was not systematically associated with hospital performance on patients achieving ≥ 25% TWL after 5 years.

## Discussion

This study demonstrates large variation between hospitals to perform specific bariatric procedures more often than would be expected given the patient-mix, suggesting a preference for that procedure. The largest number of hospitals had a preference for RYGB, only a few for OAGB. Furthermore, hospital preference for a specific type of bariatric procedure is not consistently associated with better overall weight loss outcomes for all patients treated in that hospital after 2 years; one hospital with a preference for RYGB, one hospital with a preference for OAGB, and one hospital with no preference at all had significantly more patients achieving ≥ 25% TWL after 2 years (adjusted for patient-mix). Notably, from the hospitals having a preference for SG, one hospital had significantly worse performance on achieving ≥ 25% TWL weight loss after 2 years, and one hospital with preference for RYGB performed significantly worse in patients achieving textbook outcome.

There are multiple factors that influence the choice for one bariatric procedure over another, e.g., short-term complications, long-term complications, GERD, T2D, and expected long-term weight loss [7, 8, 22]. The current study shows that patients with characteristics known to be associated with increased complications risks, such as higher ASA classification and higher BMI, were more likely to undergo SG. This is supported by literature showing lower short and long-term complication risks after SG compared to RYGB, which has led to a worldwide increase of patients undergoing SG [12]. In contrast, female patients were less likely to undergo SG [23], which has shown to be less effective in weight loss for females than males [24]. Females at child bearing age could play an important role in the decision-making process of females more often undergoing SG, given the lower postoperative complications rates compared with RYGB [2, 25]. Nevertheless, the RYGB may still be preferred in patients with T2D and GERD due to higher remission rates compared with SG [17, 26, 27]. This is also shown in the current study results with a higher likelihood to undergo RYGB for patients with T2D and GERD at baseline, whereas OAGB was preferred for patients with T2D without GERD likely due to a higher prevalence of biliary reflux [4]. Although the current study adjusted for differences in all patient characteristics, the funnel plots show various preferences for specific type of bariatric procedures between hospitals. These preferences are most likely due to (shared) surgeon preferences, which is more strongly correlated with procedure selection than patient or hospital factors [7].

Hospitals with high volume on specific bariatric procedures are associated with lower morbidity, mortality, and improved outcomes after bariatric surgery [28–31]. It has been described that performing more than 100 laparoscopic RYGB results in 50% decrease of complications [32]. Furthermore, for every 10 cases performed annually, either on hospital or surgeon level, the odds are in favor of lower major morbidity [14]. The current study shows an annual median hospital volume of 499 procedures, meaning these hospitals have procedure volumes associated with favorable outcomes. Hospitals having a preference for a specific procedure likely means they have relatively more experience with this procedure in the peri- and postoperative care process, which would suggest improved outcomes. However, the results from the present study do not show systematically better outcomes for hospitals having a preference for a specific procedure. One possible explanation could be that even though experience with a particular procedure may be slightly less in hospitals not having a preference for a specific procedure, it is still sufficient to achieve similar weight loss outcomes due to the centralized bariatric care in high volume institutions. This would also explain why hospital preference was not consistently associated with textbook outcome or CD ≥ III complications within 30 days. Of note, one hospital with RYGB preference performed significantly worse on textbook outcome, which was not due to worse peri-operative complications within 30 days, but due to their extended LOS policy of 3 days. This emphasizes the importance of the entire care process surrounding the surgery.

Long-term complications also have to be considered in shared decision making with patients to choose a specific type of bariatric procedure. After all, possible long-term complications are directly linked to the procedural technique, with possible internal herniations occurring after RYGB or OAGB, biliary reflux or malnutrition after OAGB, and GERD after SG [33]. The present study recorded 15 (0.28%), 23 (0.15%), and 2 (0.11%) complications (e.g., stricture, intestinal obstruction, gallstone, dysphagia, and internal herniation) after respectively SG, RYGB, and OAGB beyond 30 days up to 2 years of follow-up. The lower percentage complications after RYGB compared with SG are likely due to the relatively short-term follow-up as RYGB has shown to have more operative re-interventions for long-term complications up to 5 years [25].

The current study links the results of the decision-making process for procedure type to the overall hospital outcomes. A possible pitfall for hospitals with a preference could be that they also perform this procedure when perhaps another procedure might have advantages, thereby not tailoring the most suitable procedure to the clinical features of the patient as discussed previously [34]. The results for the hospital with no preference for any procedure (Fig. 3) support that bariatric patients are more likely to lose ≥ 25% of their total body weight if such a tailored choice of bariatric procedure is successful, rather than having a preference for (a) specific procedure(s) which is used on many patients [34, 35]. This shows the importance of procedure selection for the individual patient and underlines that every bariatric surgeon should be proficient in various bariatric procedures.

This study has several strengths. It includes a nationwide registry reflecting daily practice and benchmarks the quality of care after adjustment for patient-mix differences in high-volume hospitals. However, there are also limitations. Data collected as part of daily practice may be subject to errors and incomplete data. However, the mandatory design of the DATO ensures completeness and participation of all hospitals, and data verification has previously shown that the quality of entered data is reliable [36]. Second, the follow-up after 2 years was only 67%, and a longer follow-up is needed to assess long-term weight loss. However, because there was limited variation between hospitals in percentage follow-up, this is unlikely to explain the results on variation in hospital preference and their overall outcomes at 2-year follow-up. Finally, this study could not adjust for surgeon volume or surgeon preference, as no distinction between surgeons can be made from the DATO dataset. However, it seems likely that hospital preference is the result of a shared preference and hospital policy given the importance of working in teams, particularly since surgeons are collectively responsible for the outcome of their patients in the Dutch setting, as well as that all surgeons in a hospital share the work load in performing a similar number of procedures.

## Conclusion

Hospital preference for a specific bariatric procedure is not consistently associated with their overall performance on achieving ≥ 25% total weight loss for their patients after 2 years. This suggests that even though experience with a procedure may be slightly less in hospitals not having a preference, it is still sufficient to achieve similar weight loss outcomes when surgery is provided in centralized high-volume bariatric institutions.

## Supplementary Information

Below is the link to the electronic supplementary material.

ESM 1 Supplemental figure 1. Patient-mix adjusted funnel plot showing hospital variation in 25% TWL after 5 years related to preference for type of procedure (PNG 14 kb)

High resolution image (TIFF 1406 kb)

## Abbreviations

SG: Sleeve gastrectomy
RYGB: Roux-en-Y gastric bypass
OAGB: One-anastomosis gastric bypass
DATO: Dutch Audit for Treatment of Obesity
O/E: Observed/Expected
TO: Textbook Outcome
CD: Clavien Dindo
LOS: Length of stay
BMI: Body mass index
ASA: American Society of Anesthesiologists
GERD: Gastro-esophageal reflux disease
T2D: Type 2 diabetes
OSAS: Obstructive sleep apnea syndrome
