# Supplementary figures and images for: Hospital Variation in Preference for a Specific Bariatric Procedure and the Association with Weight Loss Performance: a Nationwide Analysis
Source: Obes Surg. 2022 Sep 14;32(11):3589–99. doi: 10.1007/s11695-022-06212-8 (PMC9613549; doi:10.1007/s11695-022-06212-8)

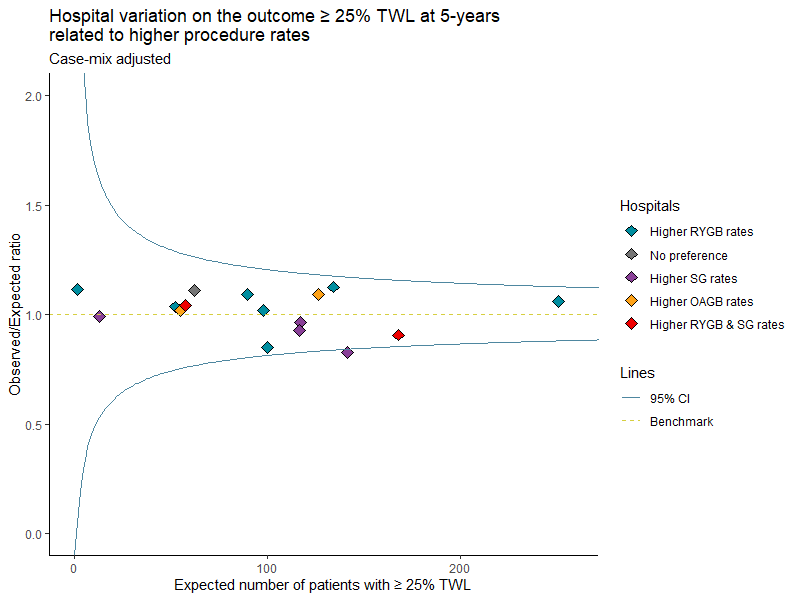

Supplement: Supplementary file 1 — Supplemental figure 1. Patient-mix adjusted funnel plot showing hospital variation in 25% TWL after 5 years related to preference for type of procedure (PNG 14 kb) [file 11695_2022_6212_Fig5_ESM.png]

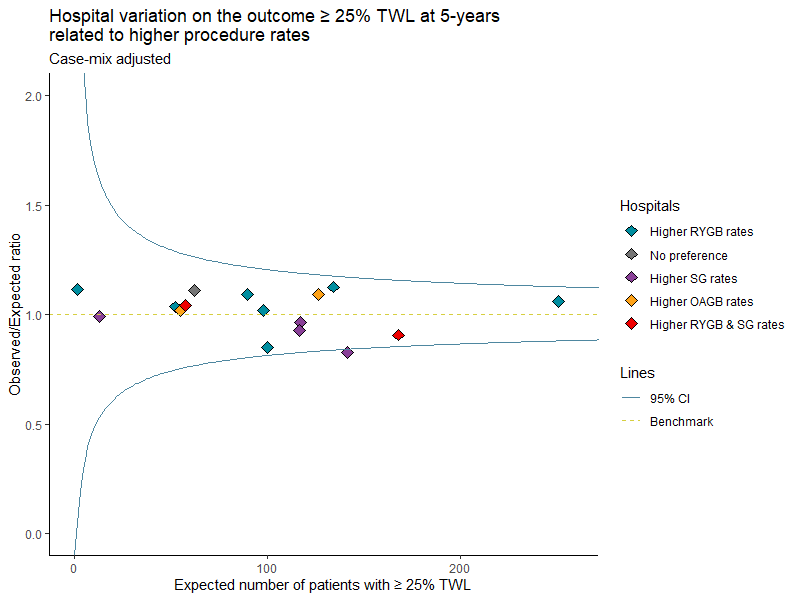

Supplement: Supplementary file 2 — High resolution image (TIFF 1406 kb) [file 11695_2022_6212_MOESM1_ESM.tiff]
